# Biomarkers for the risk of thrombosis in pancreatic adenocarcinoma are related to cancer process

**DOI:** 10.18632/oncotarget.25458

**Published:** 2018-05-29

**Authors:** Dorothée Faille, Marie-Charlotte Bourrienne, Emmanuelle de Raucourt, Luc de Chaisemartin, Vanessa Granger, Romaric Lacroix, Laurence Panicot-Dubois, Pascal Hammel, Philippe Lévy, Philippe Ruszniewski, Nadine Ajzenberg, Vinciane Rebours

**Affiliations:** ^1^ Department of Hematology, Bichat Hospital, Assistance Publique-Hôpitaux de Paris, Paris, France; ^2^ Laboratory for Vascular Translational Science (LVTS), INSERM U1148, University of Paris Diderot, Sorbonne Paris Cité, Paris, France; ^3^ Department of Hematology, Beaujon Hospital, Assistance Publique-Hôpitaux de Paris, Clichy, France; ^4^ Department of Immunology, Bichat Hospital, Assistance Publique-Hôpitaux de Paris, Paris, France; ^5^ Inflammation, Chemokines and Immunopathology, INSERM UMR 996, Paris-Sud University, Châtenay-Malabry, France; ^6^ Vascular Research Centre of Marseille (VRCM), INSERM UMR 1076, Aix-Marseille University, Marseille, France; ^7^ Haematology and Vascular Biology Laboratory, Conception Hospital, Assistance Publique-Hôpitaux de Marseille, Marseille, France; ^8^ Digestive Oncology Unit, Beaujon Hospital, Assistance Publique-Hôpitaux de Paris, Clichy, France; ^9^ Centre de Recherche sur l’Inflammation (CRI), INSERM UMR 1149, University of Paris Diderot, Sorbonne Paris Cité, Paris, France; ^10^ Department of Pancreatology and Gastroenterology, Beaujon Hospital, Assistance Publique-Hôpitaux de Paris, Clichy, France

**Keywords:** D-dimers, microparticles, pancreatic cancer, thrombosis, tissue factor

## Abstract

**Background:**

Venous thrombo-embolic events (VTE) frequently occur in patients with pancreatic ductal adenocarcinoma (PDAC) and contribute to high morbidity and mortality.

**Objectives:**

To determine whether VTE biomarkers are related to cancer, inflammation or precancerous states and to assess their relevance to predict VTE in PDAC.

**Patients and Methods:**

We compared VTE biomarkers in patients with PDAC (*n* = 42), intraductal papillary mucinous neoplasm of the pancreas (IPMN, *n* = 48) or chronic pancreatitis (*n* = 50). PDAC patients were followed-up for 6 months.

**Results:**

Factor VIII, D-dimers, von Willebrand factor, free tissue factor pathway inhibitor and microvesicle-tissue factor (MV-TF) activity were higher in PDAC patients compared to patients with IPMN or chronic pancreatitis. PDAC patients with metastasis presented higher D-dimers and MV-TF activity compared to patients with localized lesions, but elevation of D-dimers was dependent on tumor size. In multivariate analysis, elevated D-dimers (≥2.16 µg/mL) or MV-TF activity (≥2.37 pg/mL) were significant risk factors for VTE in PDAC patients, after adjustment for age and sex (HR 4.9 [1.0–23.1] or HR 10.5 [1.5–72.4], mean [interquartile range], respectively). Cumulative probability of VTE at 6 months was higher in patients with elevated D-dimers (56.3% vs 15.6%, *p =* 0.009) and in patients with high MV-TF activity (74.3% vs 21.7%, *p =* 0.01).

**Conclusions:**

VTE biomarkers including D-dimers and MV-TF activity are not related to inflammation but rather to cancer process and dissemination. D-dimers and MV-TF activity are associated to future VTE in PDAC patients and could help identify patients who could benefit from thromboprophylaxis.

## INTRODUCTION

Pancreatic ductal adenocarcinoma (PDAC) is associated with the highest incidence rate of venous thromboembolic events (VTE) in comparison to other cancer types with 14 events per 100 patients per year [1]. Hence, VTE occur in over one third of patients with PDAC [2]. VTE is observed shortly after initiation of chemotherapy [3] and, whether symptomatic or incidental, is strongly associated with higher rate of mortality [2, 4]. Of importance, a significant proportion of patients have visceral VTE such as splanchnic vein thrombosis (SVT) involving the splenic, mesenteric and/or portal veins that remain undiscovered until routine scans [2]. SVT are prognostic factors for short-term survival [5].

What makes PDAC such a thrombogenic lesion remains unclear. Vessel compression, reduced blood flow and stasis due to changes in the pancreas, together with vascular endothelial dysfunction due to inflammatory changes could explain local prothrombotic state but these pathophysiological mechanisms are not specific to cancer. Little is known about the respective roles of the neoplastic process and of the local/systemic inflammation in the prothrombotic state of patients with PDAC.

Other pancreatic diseases such as chronic pancreatitis (CP) are associated with vascular thrombotic complications. Thus, the prevalence of SVT in CP ranges from 3 to 41.7% [6]. On the other hand, although significantly higher than in the general population, the risk of thrombosis of the peripheral/pulmonary vasculature in CP patients remains lower than in PDAC patients with an overall incidence of 0.2% per year [7].

In CP patients, local inflammatory process in the pancreas appears to be the major predisposing condition for local visceral thrombosis, as inherited thrombophilia does not increase the risk of SVT [8]. Upregulated expression of tissue factor (TF), the main initiator of coagulation, in the pancreatic tissue might have a contributory role in the development of local thrombotic events in both PDAC and CP patients [9] but does not explain why thrombosis is typically found at sites distant from the primary tumor and its metastases in PDAC. The release of submicronic membrane fragments known as microvesicles (MV) from the tumor into the blood stream might be one of the mechanisms contributing to systemic blood activation in PDAC. Indeed, MV released by cultured pancreatic cancer cells exhibit TF-dependent procoagulant activity [10]. Also, cancer patients with VTE have significantly higher MV-TF activity or TF-positive MV than cancer patients without VTE [11, 12] and such a link between TF-bearing MV and VTE has been confirmed by mouse studies [13–15].

A better understanding of the pathophysiology of VTE in pancreatic cancer is of crucial importance to select biomarkers that could be useful to predict thrombotic risk in these patients and to identify ambulatory patients who could benefit from thromboprophylaxis with low-molecular-weight heparins [16]. Recently, one risk assessment model using baseline clinical (tumor entity and body mass index) and laboratory (hemoglobin level, platelet and leukocyte counts) parameters has been proposed to predict symptomatic VTE in cancer patients initiating chemotherapy [17]. The addition of biomarkers of the activation of the haemostatic system that have been associated to an increased VTE risk in patients with various types of cancer, such as soluble P-selectin and D-dimers, could improve the accuracy of this score [18, 19]. However, the performance of these risk assessment models in specific types of cancer such as PDAC and their ability to predict asymptomatic incidental VTE is still uncertain [20, 21]. Also, the cancer specificity of these biomarkers remains unknown. A comparison between PDAC and predisposing inflammatory or premalignant pancreatic diseases has not yet been performed. Only one study compared the “procoagulant profile” of PDAC and CP patients and found elevated markers of thrombin formation (thrombin-antithrombin complexes complexes or TAT, prothrombin fragments F1 + 2) in the plasma of patients with PDAC and CP compared to healthy patients [9].

Therefore, the aims of this study were to search for VTE biomarkers in patients with PDAC at various stages and to compare these results to 2 groups of patients with other types of pancreatic disease: (1) patients with CP, a pancreatic inflammatory state and (2) patients with pancreatic precancerous lesions, such as branch duct-intraductal papillary and mucinous neoplasms of the pancreas (IPMN) in order to decipher the respective roles of inflammation and neoplasia in the process of coagulation activation.

## RESULTS

### Patient characteristics

Between February 2012 and June 2014, a total of 152 patients were enrolled in the study. 12 were excluded for the following reasons: 1 patient received long term anticoagulant treatment for atrial fibrillation, 1 patient had already started chemotherapy, 1 had a primary tumor of the lung, 1 had a revised histological diagnosis of cholangiocarcinoma, 1 had a revised diagnosis of serous cystadenoma and 7 patients had no available adequate material for laboratory testing.

Fifty patients with CP, 48 patients with IPMN and 42 patients with PDAC were evaluable for procoagulant biomarkers. Baseline characteristics of the study population are presented in Table 1.

**Table 1:** Baseline demographic, clinical and biological characteristics of the total study population

	Pancreatic disease			
	CP (*n =* 50)	IPMN (*n =* 48)	PDAC (*n =* 42)	*P*-value
Age, years	47 (39–53)	65 (56–71)	66 (54–72)	<0.0001
Gender, male, *n* (%)	41 (82)	17 (35)	26 (62)	<0.0001
History of VTE, *n* (%)				
Systemic	0 (0)	0 (0)	1 (2)	NS
SVT	4 (8)	1 (2)	14 (33)	<0.0001
Leukocyte count, 10^9^/L	7.3 (13.3–9.1)	6.9 (5.5–8.0)	7.5 (6.6–8.9)	0.04
Haemoglobin, g/dL	14.1 (13.3–15.1)	13.7 (13–14.4)	13.1 (11.7–14.3)	0.002
Platelet count, 10^9^/L	233 (198–283)	232 (206–277)	212 (170–311)	NS
Fibrinogen, g/L	3.3 (2.6–4.2)	3.1 (2.9–3.4)	3.9 (3.4–4.6)	<0.0001
Interleukin-6, pg/mL	0.4 (0–2.4)	0 (0–0.8)	3.5 (0–12.3)	<0.0001
Factor VIII, %	134 (110–169)	135 (118–177)	192 (140–244)	<0.0001
TAT, ng/mL	3.6 (2.9–4.4)	3.2 (2.6–3.9)	4 (3.2–6.7)	0.003
D-dimers, µg/mL	0.38 (0.22–0.92)	0.27 (0.22–0.44)	0.91 (0.57–2.16)	<0.0001
Soluble P-selectin, ng/mL	30 (20–35)	27 (20–36)	28 (24–39)	NS
Von Willebrand factor, %	131 (87.5–161)	145 (95–178)	231 (160–392)	<0.0001
Free TFPI, ng/mL	11 (8–14)	12 (9–16)	16 (12–24)	<0.0001
Extracellular DNA, ng/mL	0 (0–11)	0 (0–5)	16 (8–34)	<0.0001
MV-TF activity, pg/mL	0.69 (0.42–0.97)	0.50 (0.38–0.80)	1.00 (0.47–2.37)	0.01
Thrombin peak, nM	194 (151–225)	226 (194–262)	214 (174–253)	0.006

CP: chronic pancreatitis, IPNM: intraductal papillary mucinous neoplasm, PDAC: pancreatic ductal adenocarcinoma, SVT: splanchnic vein thrombosis. Results are presented as number (percentage) for categorical variables or as median (IQR) for continuous variables; *p*-value for Chi-square test or Kruskal-Wallis test.

Patients with CP were significantly younger than patients with IPMN and PDAC (*p <* 0.0001). There were more men in the PC group compared to IPMN (*p <* 0.0001) and to PDAC (*p =* 0.03) groups of patients. There were also more men in the PDAC compared to IPMN group (*p =* 0.01). 33% of patients with PDAC had a previous history of SVT compared to only 8% with PC (*p =* 0.002) and 2% with IPMN (*p <* 0.0001).

### Biomarker profiles according to pancreatic disease type

To decipher the respective roles of neoplastic process and of local/systemic inflammation in the prothrombotic state of patients with PDAC, we compared the levels of various biomarkers related to coagulation or to inflammation in patients with PDAC and in two groups of patients with other types of pancreatic disease: 1/ patients with CP and 2/ patients with IPMN.

**Figure 1:**
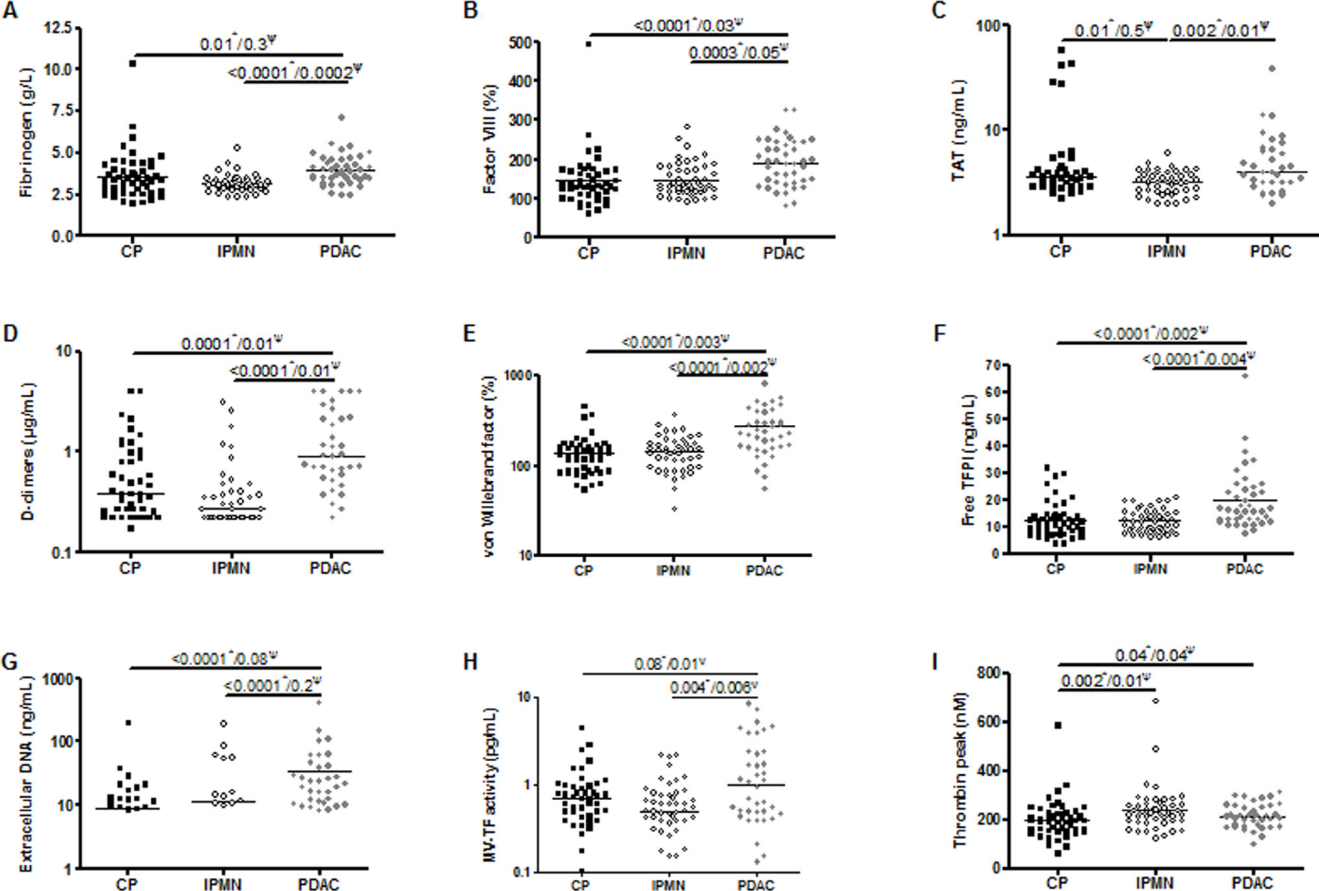
Pairwise comparison of baseline biomarker levels according to pancreatic disease subgroup: chronic pancreatitis (CP), intraductal papillary mucinous neoplasm (IPMN), pancreatic ductal adenocarcinoma (PDAC) *P*-values for ^*^Mann-Whitney test or ^Ψ^logistic regression analysis adjusted on age, sex, fibrinogen and interleukin-6 levels.

Age of patients was significantly correlated to levels of procoagulant factor VIII (*r =* 0.3, *p =* 0.001), TAT complexes (*r =* 0.2, *p =* 0.04) and VWF (*r =* 0.2, *p =* 0.008). Baseline biomarker levels according to pancreatic disease subgroup are presented in Table 1 and in Figure 1. Compared to IPMN and to CP patients, PDAC patients had significant higher levels of fibrinogen (*p <* 0.0001 and *p =* 0.01), interleukin-6 (*p <* 0.0001 and *p =* 0.009), factor VIII (*p =* 0.0003 and *p <* 0.0001), D-dimers (*p <* 0.0001 and *p =* 0.0001), VWF (*p <* 0.0001 and *p <* 0.0001), free TFPI (*p <* 0.0001 and *p <* 0.0001) and extracellular DNA (*p <* 0.0001 and *p <* 0.0001). Levels of TAT complexes and MV-TF activity were significantly higher in the PDAC group only in comparison with the IPMN group (*p =* 0.002 and *p =* 0.004, respectively). The cellular origin of circulating MV did not differ between the 3 types of pancreatic disease (Supplementary Table 1).

As fibrinogen and interleukin-6 are relevant markers of systemic inflammation, we evaluated the correlation coefficient between their levels and the other biomarkers and found a significant correlation of fibrinogen and interleukin-6 with factor VIII, D-dimers, VWF, free TFPI and extracellular DNA (Supplementary Table 2). In a regression analysis, levels of factor VIII, D-dimers, VWF, free TFPI remained significantly higher in cancer patients compared to IPMN and to CP groups, after adjustment for age, sex, fibrinogen and interleukin-6 levels (Figure 1), suggesting that the elevation of these biomarkers in PDAC patients is independent of any systemic inflammation.

### Biomarker profiles in PDAC according to metastasis

Clinical characteristics of the cancer population according to metastatic status are presented in Table 2. At the time of recruitment, metastases were found in 25 patients (59%). Locally advanced lesions were preferentially located in the pancreatic head, even if this did not reach statistical significance compared to metastatic lesions. The average size of the primary tumor was significantly larger in patients with metastatic tumors. Levels of cancer antigen 19-9 (CA 19–9) were more elevated in patients with metastatic tumors. Metastatic status was also associated with high levels of TAT complexes, D-dimers and MV-TF activity (Table 2, Supplementary Figure 1). Levels of MV measured by flow cytometry, whatever their cellular origin, were not different between patients with metastatic and patients with localized or locally advanced lesions (Supplementary Table 1).

**Table 2:** Clinical and biological characteristics of the cancer population according to the metastatic status

	No metastasis (*n =* 17)	Metastasis (*n =* 25)	*P*-value^(a/b)^
Age, years	66 (62–78)	62 (53–71)	NS
Gender, male, *n* (%)	10 (59)	16 (64)	NS
Location of tumor, *n* (%) Head Isthmus Body Tail Bifocal	11 (65) 1 (6) 4 (23) 0 (0) 1 (6)	8 (33) 1 (4) 6 (25) 5 (21) 4 (17)	NS
Median tumor size (mm)	28 (22–35)	43 (37–55)	<0.0001
CA 19-9, U/mL	112 (56–197)	1561 (242–7402)	0.008/0.09
Leukocyte count, 10^9^/L	7.2 (6.3–8.6)	7.8 (6.7–9.4)	NS
Haemoglobin, g/dL	13.1 (11.8–14.4)	13.1 (11.6–14.2)	NS
Platelet count, 10^9^/L	204 (173–337)	215 (166–311)	NS
Fibrinogen, g/L	3.6 (3.3–4.2)	4.1 (3.4–4.7)	NS
Interleukin-6, pg/mL	2.5 (0.5–18.3)	6.3 (0–12.3)	NS
Factor VIII, %	163 (134–240)	200 (149–244)	NS
TAT, ng/mL	3.2 (2.6–3.8)	5.6 (4.2–7.9)	0.006/0.1
D-dimers, µg/mL	0.6 (0.3–0.8)	1.4 (0.7–2.9)	0.004/0.1
Soluble P-selectin, ng/mL	31 (27–34)	28 (20–41)	NS
Von Willebrand factor, %	227 (147–435)	251 (160–304)	NS
Free TFPI, ng/mL	14 (11–23)	17 (13–25)	NS
Extracellular DNA, ng/mL	12 (0–34)	20 (9–35)	NS
MV-TF activity, pg/mL	0.49 (0.39–0.65)	1.69 (0.62–4.07)	0.002/0.04
Thrombin peak, nM	212 (178–256)	215 (170–253)	NS

Results are presented as median (IQR) for continuous variables or as number (percentage) for categorical variables; *p*-value for Chi-square test or ^a^Mann-Whitney test or ^b^logistic regression analysis after adjustment for tumor size.

We evaluated the correlation coefficient between tumor size or CA 19-9 levels and the other biomarkers and found a significant correlation of tumor size only with D-dimers (Spearman *r =* 0.5, *p =* 0.008). In a regression analysis, levels of D-dimers were not anymore significantly associated to metastasis after adjustment for tumor size, suggesting that their elevation in PDAC patients with metastasis is dependent on tumor size.

### Biomarker profiles in PDAC according to thromboembolic events

Forty-one of the patients with PDAC (98%) were prospectively followed for a median of 169 [IQR 68–180] days. During the 6-month observation period, 38 (93%) patients received chemotherapy, 3 (7.3%) patients were treated by radiotherapy, and 9 (22%) patients underwent pancreatic surgery. 8 patients were treated by both chemotherapy and surgery, 3 patients by both chemotherapy and radiotherapy and 2 patients by chemotherapy, radiotherapy, and surgery. 5 patients died during the follow-up.

During this follow-up period, VTE occurred in 9 (22%) patients. Clinical characteristics of the cancer population according to occurrence of VTE in the follow-up period are presented in Table 3. All patients with VTE had metastatic cancer at diagnosis. An isolated deep vein thrombosis (DVT) was diagnosed in 3, an isolated pulmonary embolism (PE) in 2, and a combined DVT and PE in 1 patient. One patient developed thrombosis of the portal vein, and one patient a thrombosis of the jugular vein. Four of the events (2 PE, thromboses of the portal vein and of the jugular vein) were detected incidentally on routine imaging. Levels of TAT complexes, D-dimers, MV-TF activity and CA19-9 were significantly higher among cancer patients with VTE (Table 3). These differences were not significant anymore if adjusted on the presence of metastasis.

**Table 3:** Demographic, clinical and biological characteristics of cancer patients according to VTE occurrence

		No VTE (*n =* 32)	VTE (*n =* 9)	*p*-value^(a/b)^
Age at study entry, years	66 (58–74)		57 (52–71)	NS
Gender, male, *n* (%)	20 (62.5)		5 (55.5)	NS
Distant metastasis, *n* (%)	15 (47)		9 (100)	0.004
Median tumor size (mm)	35 (26–49.5)		43.5 (37.5–51)	NS
CA 19-9, U/mL	194 (56–920)		7789 (229–22900)	0.03/0.1
Body Mass Index, kg/m^2^	22.0 (18.8–24.3)		22.7 (21.4–23.6)	NS
Cancer treatment, *n* (%) Chemotherapy Surgery Radiotherapy	29 (91) 9 (28) 3 (9)		9 (100) 0 (0) 0 (0)	NS NS NS
Observation time, days	180 (78–180)		94 (61–134)	0.04/1.0
Site of thrombotic event, *n* (%) Isolated DVT Isolated PE, Combined DVT + PE, Jugular vein thrombosis Brachial vein thrombosis Portal vein thrombosis			3 (37.5) 2 (25) 1 (12.5) 1 (12.5) 1 (12.5) 1 (12.5)	
Leukocytes, 10^9^/L	7.6 (6.6–8.9)		7.2 (6.6–8.8)	NS
Haemoglobin, g/dL	13.1 (11.6–14.3)		13.2 (11.65–14.6)	NS
Platelets, 10^9^/L	232 (175–330)		160 (145–262)	NS
Fibrinogen, g/L	3.9 (3.5–4.6)		3.6 (3.3–4.2)	NS
Interleukin-6 (pg/mL)	3.1 (0.2–7.6)		11 (0–14.2)	NS
Factor VIII, %	189 (140–225)		200 (154–247)	NS
TAT, ng/mL	3.5 (2.8–5.6)		6.6 (4.5–8.3)	0.02/0.8
D-dimers, µg/mL	0.71 (0.53–1.26)		2.19 (1.16–3.26)	0.04/0.2
Soluble P-selectin, ng/mL	28 (23–36)		33 (27–52)	NS
Von Willebrand factor, %	223 (158–396)		283 (167–300)	NS
Free TFPI, ng/mL	16.0 (11.7–23.5)		17.2 (14.5–25.5)	NS
Extracellular DNA, ng/mL	11.9 (4.2–28.8)		22.0 (12.5–34.2)	NS
MV-TF activity, pg/mL	0.61 (0.44–1,48)		2.23 (0.84–4.26)	0.03/0.3
Thrombin peak, nM	213 (174–244)		218 (165–264)	NS

VTE: venous thrombo-embolic event. Results presented as median (IQR) for continuous variables or as number (percentage) for categorical variables; *p*-value for ^a^Mann-Whitney test or ^b^logistic regression analysis after adjustment for metastasis.

**Figure 2:**
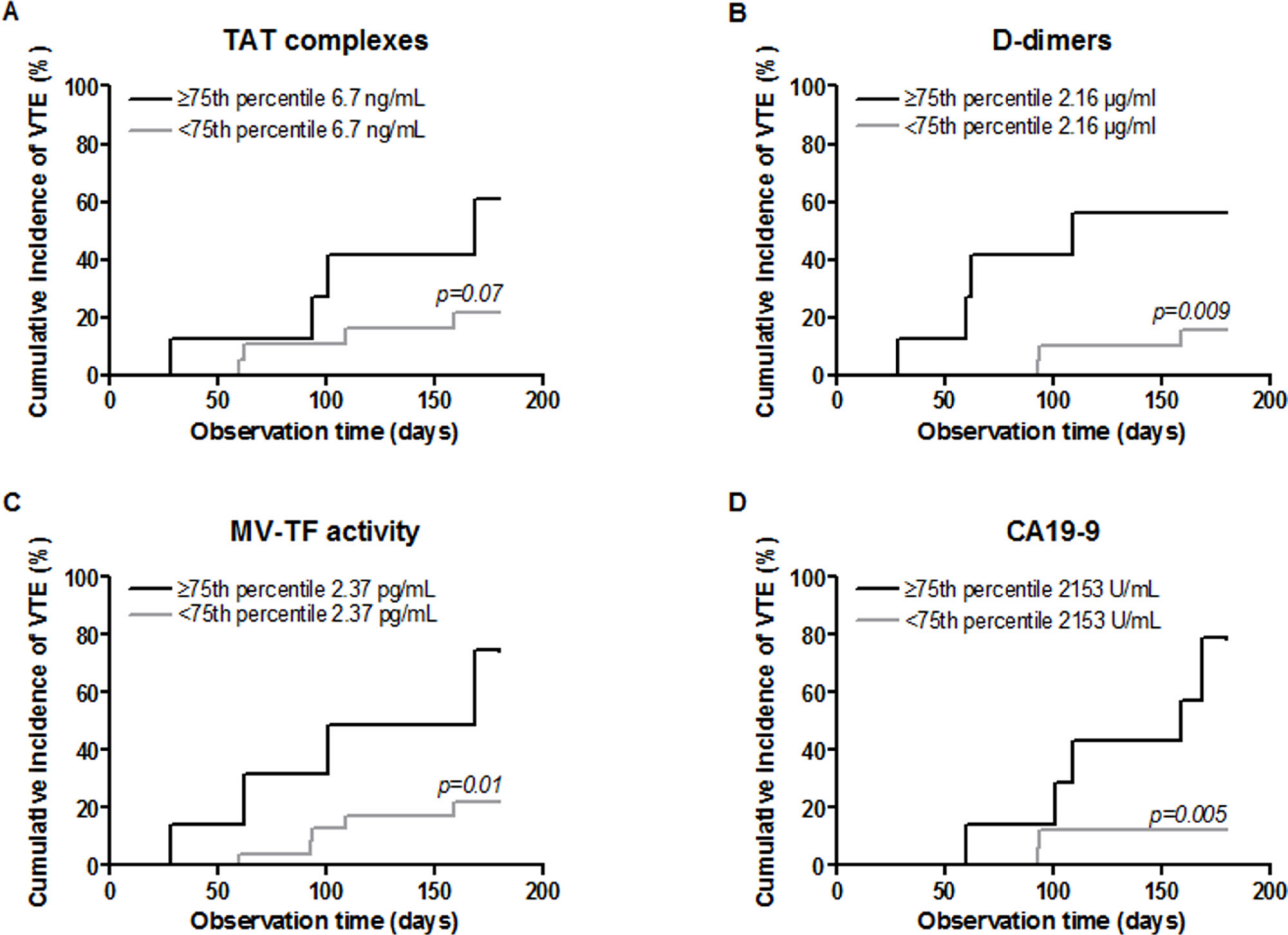
Cumulative incidence of VTE among cancer patients according to levels of D-dimers, MV-TF activity, TAT or CA-19-9 (<75th percentile or ≥75th percentile) *P*-values for log-rank test.

The risk for VTE was not statistically significant between patients with TAT levels above the 75th percentile (≥6.7 ng/mL, *n =* 8) and patients with TAT levels under the 75th percentile. The HR of VTE in patients with D-dimers levels higher than the 75th percentile (≥ 2.16 µg/mL, *n =* 8) compared with patients with levels lower than the 75th percentile was 5.8 (95% CI, 1.3–26.1, *p =* 0.02) and 4.9 (95% CI, 1.0–23.1, *p =* 0.05) in multivariate analysis including age and sex (Table 4). The cumulative probability of developing VTE after 6 months was 56.3% among patients with elevated D-dimer levels compared with 15.6% in those with lower levels (log-rank test, *p =* 0.009) (Figure 2). In patients with levels of MV-TF activity higher than the 75th percentile (≥ 2.37 pg/mL, *n =* 8), the HR for VTE was 4.6 (95% CI, 1.2–17.2), compared to patients with levels lower than the 75th percentile and 10.5 (95% CI 1.5–72.4, *p =* 0.02) after adjustment for age and sex. The cumulative probability of developing VTE after 6 months was 74.3% among patients with elevated MV-TF activity compared with 21.7% in those with lower MV-TF activity (log-rank test, *p =* 0.01). The HR of VTE in patients with levels of CA 19-9 higher than the 75th percentile (≥ 2153 U/mL, *n =* 7) compared with patients with lower levels was 7.2 (95% CI, 1.4–37.9, *p =* 0.02) and 9.5 (95% CI, 1.5–60.2, *p =* 0.02) in multivariate analysis including age and sex (Table 4). The cumulative probability of developing VTE after 6 months was 78.6% among patients with elevated levels of CA 19-9 compared with 12.5% in those with lower levels (log-rank test, *p <* 0.0001) (Figure 2).

**Table 4:** Univariate and multivariate Cox proportional hazards model for association of VTE in PDAC patients with thrombin-antithrombin complexes (TAT), D-dimers, microvesicle-tissue factor (MV-TF) activity or CA 19-9

	Univariate analysis			Multivariate analysis^*^		
	HR	95% CI	*P*	HR	95% CI	*P*
TAT, ≥ 6.7 ng/mL	3.5	0.8–13.5	0.09	4.4	0.7–29.0	0.1
D-dimers, ≥ 2.16 µg/mL	5.8	1.3–26.1	0.02	4.9	1.0–23.1	0.05
MV-TF activity, ≥ 2.37 pg/mL	4.6	1.2–17.2	0.02	10.5	1.5–72.4	0.02
CA 19-9, ≥ 2153 U/mL	7.2	1.4–37.9	0.02	9.5	1.5–60.2	0.02

^*^In multivariate analysis, each risk factor was adjusted for age and sex.

To compare the performance of biomarkers for predicting VTE in PDAC patients, we performed receiver operating characteristic (ROC) curve analysis (Supplementary Figure 3) and found that CA 19-9 had a better sensitivity and specificity than TAT complexes, D-dimers or MV-TF activity (Table 5).

**Table 5:** Receiver operating characteristic (ROC) curve analysis for the performance of thrombin-antithrombin complexes (TAT), D-dimers, microvesicle-tissue factor (MV-TF) activity and CA 19-9 in predicting VTE

	AUC	Sensitivity (%)	95% CI	Specificity (%)	95% CI
TAT, ≥ 6.7 ng/mL	0.78	84.0	63.9–95.5	50.0	15.7–84.3
D-dimers, ≥ 2.16 µg/mL	0.76	85.7	67.3–96	57.1	18.4–90.1
MV-TF activity, ≥ 2.37 pg/mL	0.74	85.7	67.3–96	44.4	13.7–78.8
CA 19-9, ≥ 2153 U/mL	0.78	90.9	70.8–98.9	71.4	29.0–96.3

AUC: area under the curve

As all patients who presented a thrombotic event during the follow-up period had a metastatic tumor at diagnosis, same analysis was performed in the subgroup of PDAC patients with metastasis (Supplementary Figure 2), the association between elevated D-dimers, MV-TF activity or CA 19-9 with VTE was not significant anymore (HR, 3.5; 95% CI, 0.8–38.8; *p =* 0.08; HR, 1.0; 95% CI, 0.2–4.8; *p =* 1.0 and HR, 2.5; 95% CI, 0.5–11.4; *p =* 0.2; respectively).

## DISCUSSION

Cancer-associated thrombosis is a major clinical problem, especially in pancreatic cancer, with no clear understanding of the pathophysiological mechanisms. Many key protagonists have been proposed to explain this high rate of thrombosis along with the development of cancer, including TF, mucins, MV, neutrophil extracellular traps, leukocytes and platelets [22]. However, the precise mechanisms of cellular activation resulting in the release of these pro-coagulant factors remain poorly studied. In the first part of this study, we attempted to clarify the respective roles of neoplastic or inflammatory processes in the pro-thrombotic state of patients with PDAC, by comparing the profile of circulating biomarkers in patients with PDAC to 2 groups of patients with other types of pancreatic disease, *i.e.* predisposing inflammatory CP or pre-invasive IPMN. We showed that fibrinogen, interleukin-6, factor VIII, D-dimers, VWF, free TFPI and extracellular DNA were higher in cancer patients than in both CP and IPMN patients. As fibrinogen and interleukin-6 are a reliable markers of systemic inflammation, we adjusted these results on fibrinogen and interleukin-6 levels and found that factor VIII, D-dimers, VWF, free TFPI and MV-TF activity were specifically elevated in PDAC patients, independently of fibrinogen and interleukin-6 levels. These results suggest that elevation of these markers was not related to systemic inflammation but rather to invasive neoplastic process. Surprisingly, markers of platelet activation, soluble P-selectin and platelet-derived MV, were not increased in PDAC patients. This is consistent with recent observations in mice. Indeed, the injection of tumor-derived MV to reach 10-fold higher plasma levels of MV-TF activity than the levels measured in pancreatic tumor-bearing mice was necessary to obtain an increase in plasma platelet factor 4 [23], suggesting that platelet activation might not be a major mechanism in the pathophysiological of PDAC-associated VTE.

In the second part of this study, to further characterize the association of biomarkers to the neoplastic process, we compared their levels between patients with metastasis and those with localized or locally advanced disease and found that patients with disseminated tumor stages had higher levels of TAT complexes, D-dimers and MV-TF activity. The role of TF in PDAC is of particular interest. TF, the principal physiologic initiator of coagulation, is expressed in pancreatic cells upon malignant transformation and its expression correlates with poor histologic grade and worsens prognosis [24–26]. In a cohort study of 73 PDAC patients, elevated MV-TF activity was present only in those with poorly differentiated metastatic non resectable tumors and was correlated with CA 19-9 and D-dimer levels [27]. MV-TF activity might thus represent a biomarker for poorly differentiated and invasive PDAC phenotype but the cellular origin of these TF-positive MV is still unclear. In a prospective cohort study on 79 PDAC patients, MV-TF activity did not correlate with the intensity of TF expression in adenocarcinoma cells but to the number of TF-positive macrophages in the surrounding stroma. These data suggest that pro-inflammatory state in the tumor environment activates macrophages expressing high amounts of TF and forming a significant source of pro-coagulant MV-TF activity [26]. Another study showed that levels of circulating TF-positive MV present in cancer patients were dramatically reduced after resection. Approximately 50% of TF-positive MV were also positive for MUC-1 antigen, suggesting that they derived from underlying malignancy [12].

In the third part of this prospective study, we analyzed the association between biomarkers and the risk of future VTE in PDAC patients and we showed that both D-dimers and MV-TF activity were associated with the occurrence of future VTE in PDAC patients. D-dimers are robust/reliable biomarkers of hypercoagulable state, as they are fibrin degradation products, reflecting both activation of coagulation and fibrinolysis and are easily measured in most routine laboratories. High D-dimer levels were proved to be associated with poor overall survival and increased mortality risk in cancer patients [28]. In PDAC patients, plasma D-dimer levels were elevated in patients with higher tumor stage, metastasis and worse differentiation grade and were associated to tumor unresectability and overall survival [29, 30]. However, their predictive value for VTE had not been established in a specific PDAC population yet.

Also, we showed that MV-TF activity was predictive of VTE in PDAC patients confirming the results of previous prospective cohort studies [26, 31, 32]. Whether the correlation of MV-TF activity with markers of coagulation activation, such as TAT complexes and D-dimers, really reflects the pro-coagulant potential of TF-positive MV remains to be elucidated. In pancreatic tumor-bearing mice, injection of exogenous TF-positive MV derived from pancreatic cancer cell lines enhanced thrombosis in a TF and thrombin-dependent manner [23, 33] and tumor-derived TF was contained in clots and increased clot size [14]. Compared to non malignant TF-positive MV, cancer TF-positive MV were able to synergize with TF from the vessel wall and to support FXa generation via the expression of phosphatidylethanolamine. Cancer TF-positive MV also altered leukocyte and platelet trafficking, resulting in thrombi primarily composed of fibrin [15]. One limitation on the use of MV-TF activity as a biomarker for VTE in clinical practice is the important source of variability and artifacts in their analysis relative to pre-analytical steps [34].

We also found that CA 19-9 levels were significantly associated to the occurrence of future VTE in PDAC patients and that the performance of CA 19-9 in predicting VTE was even better than both D-dimers and MV-TF activity. Similarly to D-dimers and MV-TF activity, the association between elevated levels of CA 19-9 and future VTE was strongly dependent on the metastatic status of patients.

Taken together, our results suggest that the hypercoagulable state observed in PDAC patients and the elevation of VTE biomarkers are related to tumor characteristics and dissemination rate. This is in line with a previous work by Woei *et al.* who showed that plasma CA19-9 levels correlated with cancer stage, short survival and with the severity of VTE [26].

Some limitations of our study should be noted. First, although our patient groups are representative of the overall population of patients with CP, IPMN or PDAC, the samples are small and heterogeneous including different tumor stages and differentiation grades. Second, patients with CP were significantly younger than patients with IPMN and PDAC and the overall differences of biomarker levels among the 3 groups of patients could be dependent on age. However, pairwise comparison of biomarkers (Figure 1) has been adjusted for this confounding factor using logistic regression analysis. Third, no routine screening test for VTE with serial ultrasonography or CT scan was performed, thus potentially underestimating the rate of incidental VTE in our cohort.

In conclusion, many biomarkers including factor VIII, D-dimers, VWF, free-TFPI and MV-TF activity are related to cancer process and elevated levels of D-dimers, MV-FT activity and CA 19-9 are risk factors for VTE in PDAC. If corroborated in further larger studies, these findings could be helpful in the identification of patients with PDAC who could benefit from thromboprophylaxis.

## MATERIALS AND METHODS

### Patients and study design

Patients with newly diagnosed pancreatic adenocarcinoma were enrolled in this prospective study in Beaujon Hospital (Clichy, France) between February 2012 and June 2014.

Two groups of patients with other types of pancreatic disease were composed according to the inclusion criteria as follows: (group 1) patients with alcoholic or non-alcoholic CP; (group 2) patients with branch duct IPMN with no worrisomes nor high risk stigmata of malignancy [35].

Exclusion criteria were defined as age <18 years, venous or arterial thrombosis within the last 6 months before inclusion, ongoing anticoagulant treatment or chemotherapy or sepsis, revised histological classification. Patients were also excluded when blood sample processing within 2 h after venipuncture was not possible. All patients underwent a structured interview on their medical history at inclusion.

This study protocol was approved by the local Ethics Committee. All patients were informed and gave written informed consent.

### Group definitions

Pancreatic adenocarcinoma diagnosis was confirmed by pathological data (pancreatic or liver metastasis biopsies) for all patients. Cancer stage was assessed by CT scan analysis.

Diagnosis of CP was based on the presence of at least one of the following: pancreatic calcifications as evidenced by CT scan or endoscopic ultrasonography, moderate to marked pancreatic ductal lesions on pancreatography obtained by endoscopic retrograde or magnetic resonance pancreatography (Cambridge classification) [8]. All causes of CP were considered.

Branch duct IPMN diagnosis relied on one or several branch duct dilatation(s) with normal main pancreatic duct, or pancreatic cystic lesions communicating with pancreatic ducts on at least two imaging techniques (MRI, CTscan or endoscopic ultrasound). Patients with no worrisomes nor high risk stigmata features were selected according to the SENDAI guidelines [35].

### Data collection

The following clinical data were collected at inclusion: body mass index, tobacco and alcohol consumption, dyslipidemia, diabetes mellitus, arterial hypertension, past medical history of venous or arterial thrombosis and consequent antithrombotic therapy used.

For patients with PDAC, presence and location of metastasis at inclusion, chemotherapy protocol and number of lines during follow-up were collected.

Follow-up period started at the time of inclusion and a regular follow-up was performed every 3 months regarding the occurrence of VTE. The observation period ended after a 6-month period or until the occurrence of VTE, death or loss of follow up. No systematic screening for VTE was performed during the follow-up. All symptomatic VTE were confirmed by either doppler sonography, venography, or CT-scan. Incidentally discovered VTE (eg, pulmonary embolism or visceral thrombosis detected in a routine CT-scan) were also considered.

### Blood sampling

Venous blood samples were drawn into 3.2% (0.109M) citrate vacuum tubes (Vacuette^®^, 3.5 mL, Greiner-Bio One). The first 5 milliliters of blood were discarded. All samples were processed within 2 hours. Whole blood samples were centrifuged once at 2500 g for 15 min at room temperature (RT). From each tube, the platelet-poor plasma was collected and was then centrifuged a second time at 2500 g for 15 min at RT to obtain platelet-free plasma (PFP). PFP aliquots were stored at –80° C until analysis.

### Laboratory measurements

Fibrinogen (Dade^®^ Thrombin reagent, Siemens, Marburg, Germany) and coagulation factor VIII (TriniCLOT automated aPTT, Diagnostica Stago, Asnières, France) were determined by chronometric coagulation assays on BCS (Dade Behring, Deerfield, IL, USA). D-dimer levels were measured by quantitative latex assay (STA-Liatest D-Di^®^, Diagnostica Stago) on a STA-R analyzer (Diagnostica Stago). Soluble P-selectin (R&D Systems, Minneapolis, MN, USA), interleukin-6 (R&D Systems), von Willebrand factor antigen (Asserachrom^®^ VWF:Ag, Diagnostica Stago) and free TFPI (Asserachrom^®^ Free TFPI, Diagnostica Stago) were measured by immunoassay following the manufacturer’s instructions. Circulating extracellular DNA was extracted from plasma samples using QIAamp^®^ UltraSens Virus Kit (Qiagen) and measured using the PicoGreen Quant-iT dsDNA Assay Kit (Life Technologies). The measurement of MV-TF activity was performed according to standardized protocol for a chromogenic endpoint assay measuring TF-dependent Xa generation as reported previously [36]. Plasma thrombin generation was measured in PFP using the Calibrated Automated Thrombogram (CAT^®^, Diagnostica Stago) at a final concentration of 1 pM TF and 4 mM phospholipids (PPP-Reagent LOW^®^, Diagnostica Stago).

### Measurement of circulating MV by flow cytometry

Circulating MV were labelled directly in PFP with fluorescein isothiocyanate (FITC)-conjugated annexin V (Beckman Coulter) and one of the following phycoerythrin (PE)-conjugated mouse monoclonal antibody to specific human cell surface markers : anti-platelet CD41 (IgG1, clone P2, Beckman Coulter), anti-erythrocyte CD235a (glycophorin A, IgG1, clone 11E4B-7-6, Beckman Coulter), anti-leukocyte CD15 (IgM, clone 80H5, Beckman Coulter), anti-endothelial CD144 (VE-cadherin, IgG1, clone TEA 1/31, Beckman Coulter), anti-epithelial CD227 (MUC1, IgG1, clone VU4H5, Santa Cruz) or corresponding isotype control (IgG1, clone 679.1Mc7, Beckman Coulter). MV were analyzed by flow cytometry with an EPICS XL (Beckman Coulter) over a 60 sec period of time. 0.9-µm latex beads (Diagnostica Stago) were used to calibrate MV size. MV absolute numbers were calculated using a flow rate based calibration method.

### Statistical analysis

Categorical variables are presented as number of subjects with percentage. Continuous variables are presented as median with interquartile range (25th-75th percentiles). Comparisons between the 3 groups were made using Kruskal-Wallis test for continuous variables and chi-square test for categorical variables. Post-hoc pairwise comparison of biomarkers was performed using Mann-Whitney *U* test and a logistic regression analysis was used to adjust for age, sex, fibrinogen and interleukin-6 levels. Cancer patients were then further categorized according to metastasis; comparisons of biomarkers were done by Mann-Whitney *U* test and a logistic regression analysis was used to adjust for tumor size. Univariate correlation between continuous variables was performed by Spearman correlation. Each biomarker level was analyzed as a continuous variable and as a binary variable with a cut-off value set at the 75th percentile of the cancer population. Univariate and multivariate Cox regression analyses were used for calculating the risk of VTE from study inclusion until first VTE, loss to follow-up, patient’s death or maximal length of follow-up of 6 months (end of the study). The analyses were adjusted for age at study inclusion and sex. Kaplan-Meier analysis was used to visualize the risk of developing VTE in PDAC patients with biomarker levels below and above the threshold. A log-rank test was used to assess differences in developing VTE in these two groups. The predictive performance of biomarkers for VTE was evaluated using ROC curve analysis. Sensitivities and specificities were calculated using a cut-off value set at the 75th percentile of the cancer population. *P*-values smaller than 0.05 were regarded as statistically significant. All analyses were performed using Statview^®^ 5.0. (SAS Institute Inc, Cary, NC, USA).

## SUPPLEMENTARY MATERIALS FIGURES AND TABLES


