# Supplementary material for: Biomarkers for the risk of thrombosis in pancreatic adenocarcinoma are related to cancer process
Source: Oncotarget. 2018 May 29;9(41):26453–65. doi: 10.18632/oncotarget.25458 (PMC5995170; doi:10.18632/oncotarget.25458)
Supplement: Supplementary file 1 [file oncotarget-09-26453-s001.pdf]

Biomarkers for the risk of thrombosis in pancreatic adenocarcinoma are related to cancer process

SUPPLEMENTARY MATERIALS

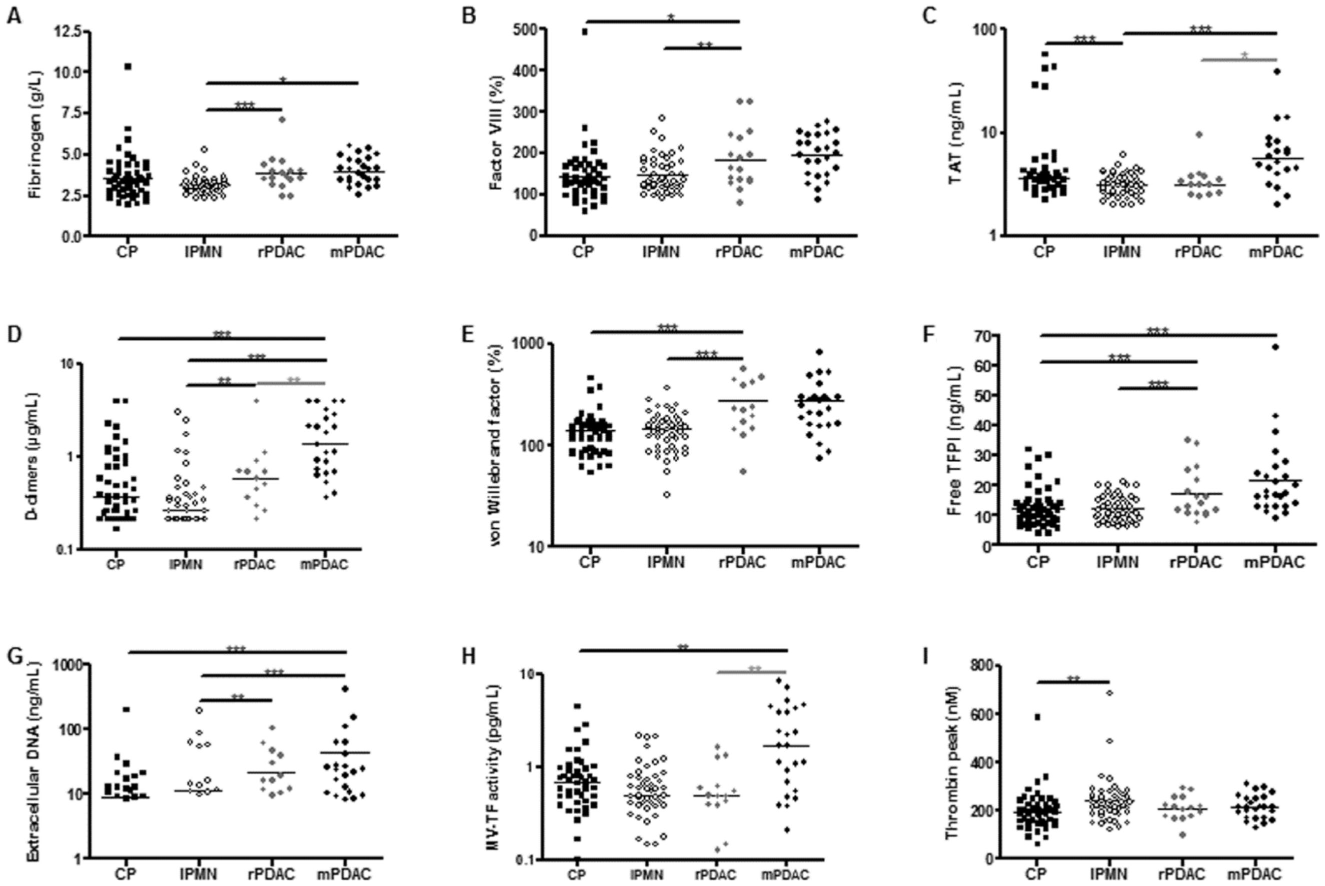

Supplementary Figure 1: Baseline biomarker levels according to pancreatic disease subgroup: chronic pancreatitis (CP), intraductal papillary mucinous neoplasm (IPMN), localized resectable pancreatic ductal adenocarcinoma (rPDAC) or metastatic pancreatic ductal adenocarcinoma (mPDAC). Statistically significant *p*-values for Mann-Whitney test: $^{*} < 0.05$; $^{**} < 0.01$ or $^{***} < 0.001$.

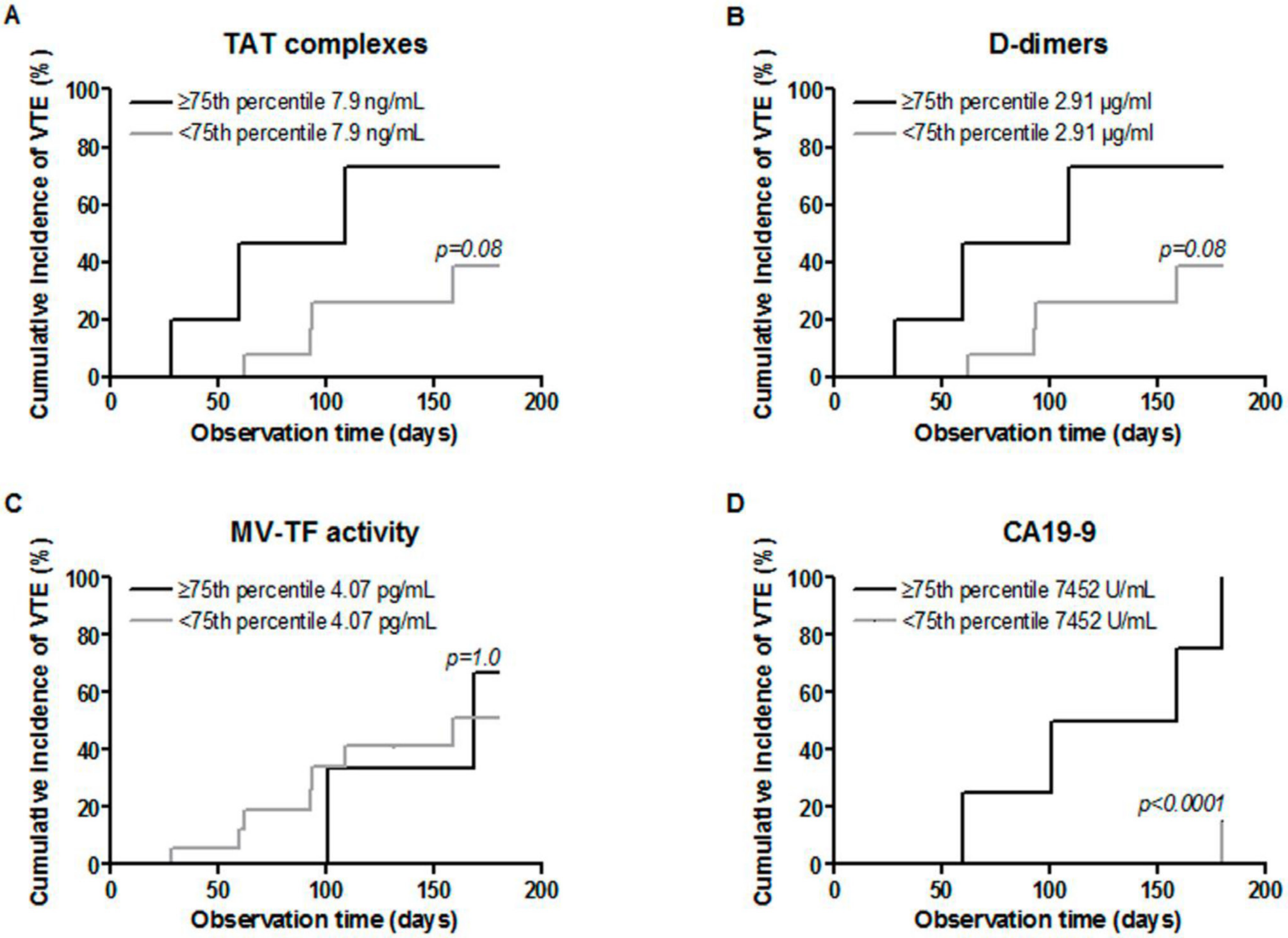

Supplementary Figure 2: Cumulative incidence of VTE among cancer patients with metastasis at diagnosis according to levels of TAT complexes (**A**), D-dimers (**B**), MV-TF activity (**C**) or CA 19-9 (**D**) (<75th percentile or ≥75th percentile).

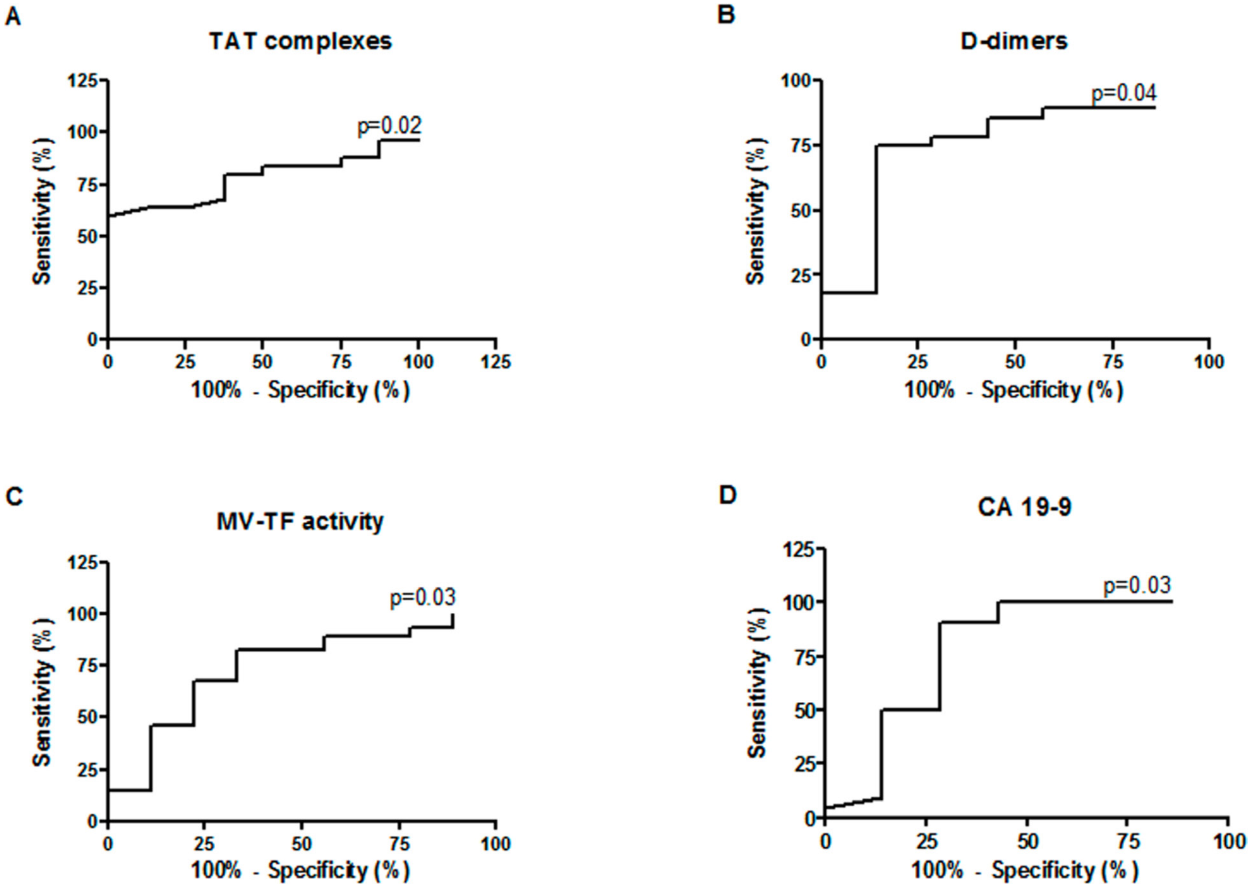

Supplementary Figure 3: Receiver operating characteristic (ROC) curve analysis for the performance of TAT complexes (**A**), D-dimers (**B**), MV-TF activity (**C**) or CA 19-9 (**D**) in predicting VTE in PDAC patients.

Supplementary Table 1: Characterization of plasma microvesicle (MV) subpopulations in patients with chronic pancreatitis (CP), intraductal papillary mucinous neoplasm (IPMN), localized resectable pancreatic ductal adenocarcinoma (rPDAC) or metastatic pancreatic ductal adenocarcinoma (mPDAC)

	Pancreatic disease				
	**CP (*n* = 50)**	**IPMN (*n* = 48)**	**rPDAC (*n* = 17)**	**mPDAC (*n* = 25)**	***P*-value**
Total procoagulant MV (annexin V+)	3922 (1693–8400)	2597 (1950–3663)	2590 (1255–11423)	4230 (1489–18100)	0.210
Platelet MV (annexin V+/CD41+)	1957 (966–5245)	1738 (1073–2450)	1502 (559–7765)	2339 (918–11960)	0.398
Erythrocyte MV (annexin V+/CD235a +)	506 (230–762)	376 (211–652)	264 (95–598)	406 (207–916)	0.183
Leukocyte MV (annexin V+/CD15+)	31 (14–76)	26 (13–46)	28 (10–67)	47 (12–88)	0.388
Endothelial MV (annexin V+/CD144+)	28 (14–59)	20 (12–41)	18 (11–65)	25 (15–81)	0.334
Epithelial MV (annexin V+/MUC-1+)	165 (42–384)	99 (49–165)	82 (41–279)	221 (41–977)	0.167

Results presented as median levels of MV/µL (IQR); *p*-value for Kruskal-Wallis test.

Supplementary Table 2: Correlation of fibrinogen and interleukin-6 with the other biomarkers

	Fibrinogen		Interleukin-6	
	**Spearman r**	***P*-value**	**Spearman r**	***P*-value**
Leukocyte count	0.2	0.004	0.2	0.004
Haemoglobin,	–0.1	NS	–0.2	0.03
Platelet count,	0.2	0.05	–0.05	NS
Fibrinogen	–	–	0.4	<0.0001
Factor VIII	0.2	0.003	0.3	<0.0001
TAT	0.3	0.0001	0.09	NS
D-dimers	0.3	0.0003	0.3	0.0001
Soluble P-selectin	0.04	NS	0.1	NS
Von Willebrand factor	0.3	<0.0001	0.4	<0.0001
Free TFPI	0.2	0.02	0.2	0.006
Extracellular DNA	0.3	0.0006	0.3	0.003
TF-MP activity	0.1	NS	0.04	NS
Thrombin peak	0.04	NS	0.05	NS
